# Commentary: An engineered and scalable human cell-derived extracellular matrix demonstrates advantages over recombinant collagen in biomimicry and wound healing

**DOI:** 10.3389/fbioe.2026.1986764

**Published:** 2026-10-27

**Authors:** Zhengrui Liu, Lei Cui, Mengcheng Yao, Jinming She

**Affiliations:** 1 Department of Medical Aesthetics, Affiliated Central people’s Hospital, China Three Gorges University, Yichang, Hubei, China; 2 Yichang Central People’s Hospital, Yichang, China

**Keywords:** extracellular matrix, fibroblast, p16, p21, wound healing

## Introduction

We read this article with great interest. Guo et al. (2026) proposed an engineered and scalable human cell-derived extracellular matrix (HE-ECM), which can promote wound healing. This paper successfully established a large-scale, engineered preparation strategy for human fibroblast extracellular matrix (ECM) and demonstrated, through systematic *in vitro* and *in vivo* experiments, that HE-ECM has significant advantages in biomimetic properties, biological activity, and promoting wound healing. We fully agree with the conclusions of this article and recognize its significant clinical value. In addition to the factors already discussed in the original article, changes in cell state during the *in vitro* culture stage, such as senescence, also affect the production of ECM. In the original paper, the use of commercial fibroblasts at passages 3–5 was emphasized, but detection of cellular senescence markers was still suggested. This is because cellular senescence and related phenotypic changes occur and manifest as senescence-associated secretory phenotype (SASP)-mediated paracrine effects (Bihuniak et al., 2026).

## Senescence-associated cytotoxic responses

Previous studies have reported that *in vitro* cultivation of fibroblasts faces an inevitable challenge: abnormalities in cellular states such as senescence can lead to a decrease in cellular activity, as manifested by increased expression of the cyclin-dependent kinase inhibitors p16 (CDKN2A) and p21 (CDKN1A), increased senescence-associated β-galactosidase (SA-β-gal) activity, and significant changes in the ECM expression profile (Di Micco et al., 2021; Wang et al., 2024). The expression levels of mRNAs encoding collagen type I α1 and type III α1 were lower in senescent fibroblasts (Shelton et al., 1999; Mavrogonatou et al., 2019; Boaru et al., 2025). Furthermore, studies have indicated that senescent fibroblasts possess a pro-inflammatory and pro-fibrotic secretory profile, exerting detrimental effects (Wang et al., 2024; Xue et al., 2022). Moreover, stem cell-derived extracellular vesicles (SC-EVs) belong to the senomorphic rather than the senolytic (senescent cell-clearing) class of anti-aging therapeutic agents. These vesicles, by delivering cargo such as proteins and miRNAs, act on fibroblasts, inhibiting the senescent phenotype and suppressing SASP-associated effects (Rudnitsky et al., 2024; Zhang et al., 2025).

## Discussion

The original study demonstrates that HE-ECM, owing to its retention of the complex protein components and three-dimensional porous structure of the native matrix, is superior to recombinant collagen in promoting fibroblast activity and accelerating wound healing. However, the cytotoxic effects arising from cellular senescence cannot be overlooked. These observations suggest that the senescence affects the functionality of scalable, engineered HE-ECM. Selecting reliable and robust detection markers is essential for stable quality control of this approach. The engineered HE-ECM strategy proposed by the original authors, which utilizes a commercial human fibroblast cell line and a standardized decellularization process, enables scalable production of ECM with stable composition and enrichment of multiple matrix proteins (such as collagen, fibronectin, proteoglycans, and matrix-remodeling enzymes), showing promising clinical translation potential. The experimental measurements of fibroblast population doubling level (PDL) and senescence phenotype markers (p16, p21, SA-β-gal, etc.) should be performed (Di Micco et al., 2021; Alcorta et al., 1996; Dimri et al., 1995). Taken together, the possibility cannot be excluded that cellular senescence may alter the ECM expression profile and impair reparative capacity. PDL/passage number at the point of ECM harvest, inclusion of senescence marker panels as batch-release quality criteria, and correlation of senescence marker levels with downstream ECM functional outcomes should be addressed.
